# Ammonium Accumulation Caused by Reduced Tonoplast V-ATPase Activity in *Arabidopsis thaliana*

**DOI:** 10.3390/ijms22010002

**Published:** 2020-12-22

**Authors:** Guihong Liang, Haixing Song, Yan Xiao, Zhenhua Zhang

**Affiliations:** Southern Regional Collaborative Innovation Center for Grain and Oil Crops in China, College of Resources and Environmental Sciences, Hunan Agricultural University, Changsha 410128, China; ghliang1119@hunau.edu.cn (G.L.); hxsong@hunau.edu.cn (H.S.); yanx@hunau.edu.cn (Y.X.)

**Keywords:** proton pump, vacuole, nitrate, ammonium, ion homeostasis, potassium

## Abstract

Plant vacuoles are unique compartments that play a critical role in plant growth and development. The vacuolar H^+^-ATPase (V-ATPase), together with the vacuolar H^+^-pyrophosphatase (V-PPase), generates the proton motive force that regulates multiple cell functions and impacts all aspects of plant life. We investigated the effect of V-ATPase activity in the vacuole on plant growth and development. We used an *Arabidopsis* *thaliana* (L.) Heynh. double mutant, *vha-a2 vha-a3*, which lacks two tonoplast-localized isoforms of the membrane-integral V-ATPase subunit VHA-a. The mutant is viable but exhibits impaired growth and leaf chlorosis. Nitrate assimilation led to excessive ammonium accumulation in the shoot and lower nitrogen uptake, which exacerbated growth retardation of *vha-a2 vha-a3*. Ion homeostasis was disturbed in plants with missing *VHA-a2* and *VHA-a3* genes, which might be related to limited growth. The reduced growth and excessive ammonium accumulation of the double mutant was alleviated by potassium supplementation. Our results demonstrate that plants lacking the two tonoplast-localized subunits of V-ATPase can be viable, although with defective growth caused by multiple factors, which can be alleviated by adding potassium. This study provided a new insight into the relationship between V-ATPase, growth, and ammonium accumulation, and revealed the role of potassium in mitigating ammonium toxicity.

## 1. Introduction

The evolutionary success of higher plants is largely contributed to their unique cellular architecture [1]. Vacuoles, as the largest organelles in mature plant cells, accounting for about 90% of the cell volume, contain a substantial amount of water and solutes necessary for plant growth and development [2,3,4]. The transport of molecules across the tonoplast is driven by the proton gradient and membrane potential established by the combined activity of two proton pumps, the vacuolar H^+^-ATPase (V-ATPase) and vacuolar H^+^-pyrophosphatase (V-PPase), both of which are essential for plant growth and development [1,5].

V-ATPase and V-PPase use Mg-ATP and Mg-inorganic pyrophosphate (PPi) complexes, respectively, as substrates [6]. Through the combined activity of the two pumps, protons enter the vacuole from the cytoplasm and an electrochemical gradient is established between the cytoplasm and vacuole, which drives the transport of compounds across the tonoplast against their concentration gradient [1]. Both proton pumps are among the most abundant tonoplast proteins, thereby indicating that the amount of energy spent in vacuolar transport is enormous [7,8]. V-ATPase is structurally conserved and consists of two sub-complexes. The peripheral V_1_ complex is composed of eight subunits (VHA-A to -H), which are exposed to the cytoplasmic side and are responsible for ATP hydrolysis. The membrane-integral V_0_ complex consists of six subunits (VHA-a, -c, -c’, c’’, -d, and -e), which are integrated into the membrane and act as a channel for translocation of protons from the cytoplasm into the lumen of endomembrane compartments [9]. This complex also serves as the binding site for polymerization and assembly of V_1_ subunits [9,10].

In *Arabidopsis thaliana* (L.) Heynh, three isoforms of the integral membrane subunit VHA-a have different subcellular locations: VHA-a1 is located in the trans-Golgi network/early endosome (TGN/EE), while VHA-a2 and VHA-a3 are located in the tonoplast [11]. The *Arabidopsis thaliana* double mutant *vha-a2 vha-a3,* which lacks the two tonoplast-localized isoforms of the membrane-integral V-ATPase subunit VHA-a, is viable but exhibits symptoms of severe growth inhibition and premature senescence [4].

There are multiple types of ion transporters and channels in the tonoplast that transport NO_3_^−^ [12], Na^+^ [13,14], and many other ions [15,16]. The activity of proton pumps in the tonoplast provides a driving force for the active transport of various solutes. Altered activities of either of these two proton pumps significantly influence vacuolar transport capacities to regulate metabolite homeostasis, plant growth, and plant adaptation to abiotic stress [7,12,17,18,19,20]. After its absorption by plants, nitrate, an important plant nutrient, is allocated to the metabolic pool (cytoplasm) and the storage pool (vacuole) [21]. The change in relative size of the two nitrate reservoirs is mediated by the NO_3_^−^/H^+^ exchange channel CLC-a in the tonoplast and the activity of V-ATPase and V-PPase [4,22]. Ion homeostasis and pH depend on the coordinated action of multiple transporters at the plasma membrane (PM) and in subcellular organelles, such as the vacuolar membranes [23,24]. The cytoplasmic pH of plant cells is regulated within narrow limits, usually at about pH 7 to 7.5 [24]. Together, proton (H^+^) and ion pumps, cotransporters, and channels determine ionic cellular distribution, which is critical for the maintenance of membrane potential, pH control, protein activity, and transport of nutrients [25].

Potassium (K^+^) is one of the most important nutrients required by plants [26]. K^+^ is absorbed by plant roots, and its transport is mediated by K^+^ channels and high-affinity K^+^ transporters [27,28] in both the plasma membrane and the tonoplast of plant cells [25,29,30]. Emerging evidence continues highlight interactions between K^+^ and other nutrients, such as NO_3_^−^ and Pi [31,32,33,34,35], which affect the plant growth. In addition to being an essential nutrient in plants, K^+^ is crucial for enzyme activities and ion homeostasis [36,37]. Vacuolar H^+^-PPase, another main proton pump in the tonoplast, is stimulated by potassium [38]. K^+^ also alleviates the growth inhibition caused by ammonium toxicity; an increased K^+^ supply or restoration of K^+^ transport can rapidly relieve NH_4_^+^ toxicity [39,40].

A decreased proton pump activity affects the growth and development of plants [1,4]; however, the contributing factors and metabolic mechanisms remain unknown. The aim of the present study was to investigate (a) the effect of V-ATPase on growth and development of plants using a double mutant lacking the V-ATPase activity in the tonoplast, and (b) the effect of potassium supplementation on ammonium accumulation. Here, we showed that the *Arabidopsis thaliana* double mutant *vha-a2 vha-a3*, which lacks the two tonoplast-localized isoforms of the membrane-integral V-ATPase subunit VHA-a, was viable but with impaired growth and excessive ammonium accumulation, which led to premature senescence of the mutant plants. The growth inhibition of the double mutant was alleviated, senescence delayed, and content of ammonium decreased in mutant plants grown in the presence of supplemental K^+^. Our results provide new insights into the relationship between V-ATPase, growth, and ammonium accumulation. The phenotype of the *vha-a2 vha-a3* mutant showed the effect of potassium on internal ammonium content through mitigation of the activity of tonoplast V-ATPase.

## 2. Results

### 2.1. V-ATPase Activity at the Tonoplast Regulates Growth and Development

**Figure 1:**
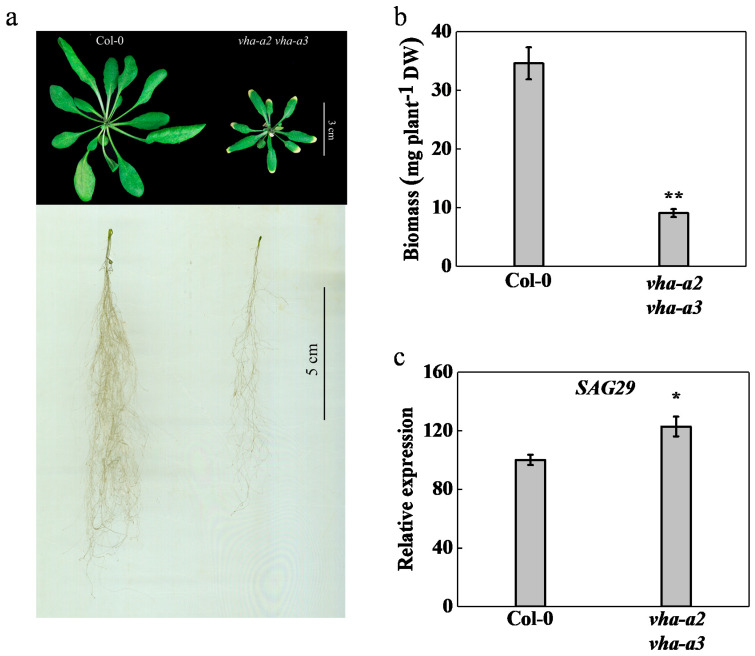
Loss of tonoplast vacuolar H^+^-ATPase (V-ATPase) caused severe growth inhibition and senescence. (**a**) phenotype comparison between *Arabidopsis thaliana* wild type (Col-0) and the *vha-a2 vha-a3* double mutant. (**b**) the biomass of Col-0 and *vha-a2 vha-a3* was determined. (**c**) gene expression of senescence-associated gene 29 (*SAG29)* in wild type and *vha-a2 vha-a3* four-week-old leaves. Wild type was set to 100% and error bars were defined as S.E. of *n* = 3 biological replicates. Pictures show four-week-old plants in hydroponics. Error bars in (**b**) indicate S.D. of *n* = 3 technical replicates. Asterisks (*) and (**) indicate significant differences at *p* < 0.05 and *p* < 0.01, respectively.

The *vha-a2 vha-a3* double mutant exhibited severe growth retardation (biomass was reduced by 74%) and significant symptoms of premature aging compared with those of the wild type (Figure 1a). The relative expression of senescence-associated gene 29 (*SAG29*) was upregulated in the mutant when grown hydroponically for four weeks (Figure 1b,c). Compared with those of the wild type, leaf area, total root length, root surface area, root volume, and the number of root tips and forks were reduced by 58%, 61%, 82%, 90%, 37%, and 71%, respectively (Figure S1). Accordingly, plants exhibited poor growth in the absence of *VHA-a2* and *VHA-a3*.

### 2.2. Loss of Tonoplast V-ATPase Reduces Nitrogen Absorption

**Figure 2:**
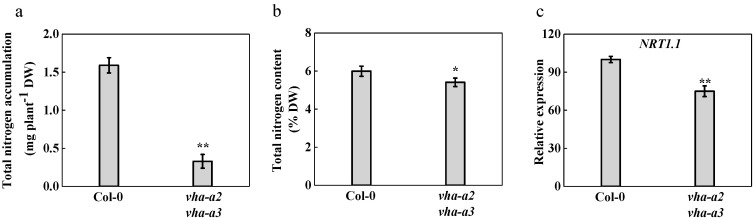
Nitrogen absorption in wild type and the *vha-a2 vha-a3* double mutant. Total nitrogen accumulation (**a**) and total nitrogen content (**b**) were measured of wild type and *vha-a2 vha-a3* mutant. Data in (**a**) and (**b**) were mean ± S.D. (*n* = 3). (**c**) Relative expression of nitrate transporter 1.1 (*NRT1.1*) in Col-0 and *vha-a2 vha-a3* was measured and Col-0 was set to 100%. Data in are mean ± S.E. (*n* = 3). Asterisks (*) and (**) indicate significant differences at *p* < 0.05 and *p* < 0.01, respectively. DW, Dry weight.

To investigate whether the absence of tonoplast V-ATPase activity would restrain nitrogen absorption and inhibit plant growth, the total nitrogen accumulation and total nitrogen content of the *vha-a2 vha-a3* double mutant were determined (Figure 2a,b). Total nitrogen content was significantly reduced by 9.7% compared with that of the wild type. Additionally, the relative expression of *nitrate transporter 1.1* (*NRT1.1*) gene was downregulated by 25% in the tonoplast of the V-ATPase-deficient double mutant (Figure 2c). These data suggest that the severe growth inhibition of the *vha-a2 vha-a3* mutant may be caused by the reduced total N uptake.

### 2.3. Disruption of Nitrogen Metabolism Causes Ammonium Accumulation in Shoots of Mutant Plants

**Figure 3:**
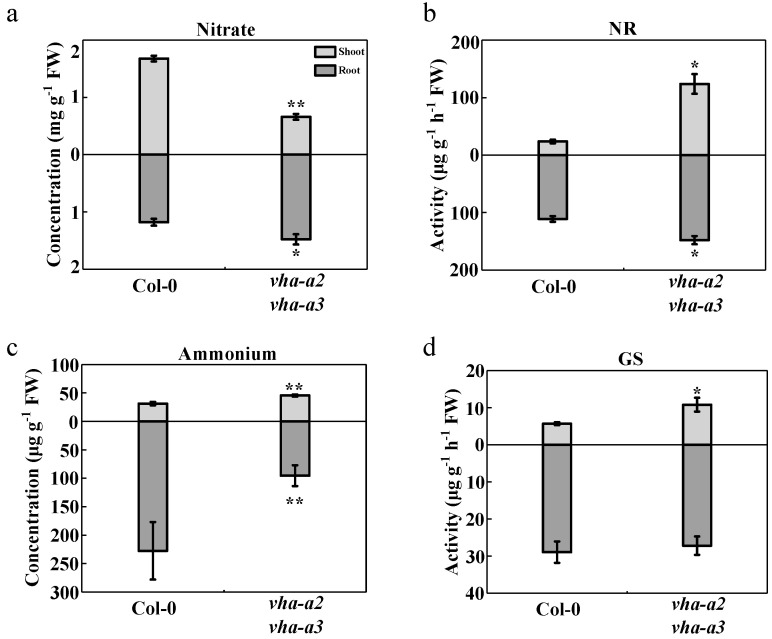
Nitrogen metabolism was measured using wild type and *vha-a2 vha-a3* mutant four-week-old seedlings in hydroponics. Nitrate concentration (**a**), nitrate reductase (NR) activity (**b**), ammonium concentration (**c**), and glutamine synthetase (GS) activity (**d**) of Col-0 and *vha-a2 vha-a3* mutant were determined in the shoots and roots. Error bars represent S.D. of three biological replicates. Asterisks (*) and (**) indicate significant differences at *p* < 0.05 and *p* < 0.01, respectively. FW, Fresh weight.

Altered activity of the proton pump significantly influences nitrate accumulation within the vacuole [12], affecting the nitrogen metabolism. With respect to the wild type, the *vha-a2 vha-a3* mutants contained 61% less nitrate (Figure 3a) and nitrate reductase (NR) activity in shoots increased four-fold (Figure 3b). Moreover, the content of ammonium in the double mutant increased by 45% compared with that in the wild type (Col-0) (Figure 3c), and the levels of glutamine synthetase (GS) increased (Figure 3d). Chlorophyll content, as an indicator of ammonium accumulation, was significantly higher in the double mutant than in Col-0 (Figure S2A), indicating that the *vha-a2 vha-a3* double mutant was possibly exposed to mild ammonium stress [41].

### 2.4. Absence of Tonoplast V-ATPase Affects the Long-Distance Transport of Nitrogen between Shoots and Roots

**Figure 4:**
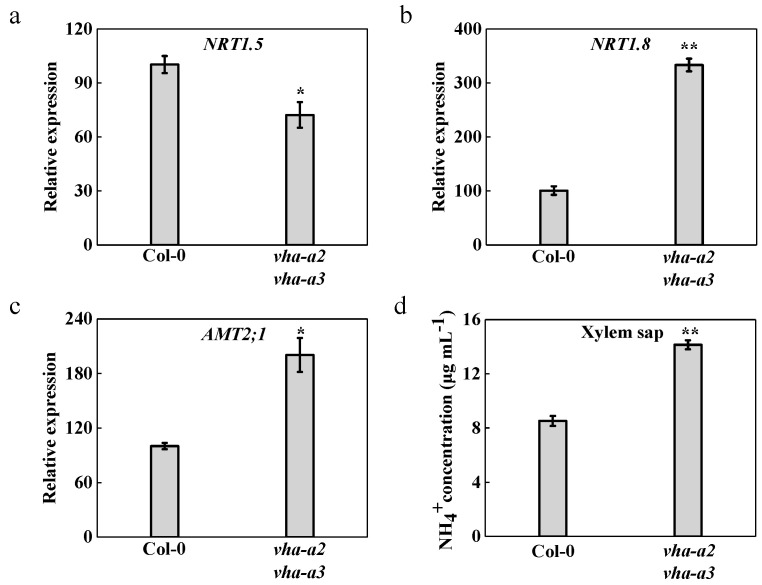
Nitrogen transport over long distance between shoots and roots. Relative expression of *NRT1.5* (**a**), *NRT1.8* (**b**), and ammonium transporter (AMT)2*;1* (**c**) was determined from the roots of wild type and *vha-a2 vha-a3* mutant. The wild type was set to 100%. (**d**) higher xylem sap NH_4_^+^ concentration in *vha-a2 vha-a3* mutant. Error bars in (**a**) to (**c**) were mean ± S.E. (*n* = 3) and in (**d**), mean ± S.D. (*n* = 3). Asterisks (*) and (**) indicate significant differences at *p* < 0.05 and *p* < 0.01, respectively.

A proportion of the NO_3_^−^ absorbed by plant roots is stored within vacuoles or assimilated into organic nitrogen through a series of reactions catalyzed by NR, nitrite reductase, GS, and glutamate synthase [42]. However, most of absorbed NO_3_^−^ is primarily transported upward through the xylem and downward through the phloem [43]. The long-distance transport and distribution of NO_3_^−^ between the roots and shoots in *Arabidopsis thaliana* are mainly co-regulated by *NRT1.5* and *NRT1.8*, two members of the peptide transporter (PTR) family, the expression of which is strongly induced by NO_3_^−^ [44,45,46,47,48]. In a wide range of plants, the transport of ammonium across membranes is mediated by proteins of the ammonium transporter/methylammonium permease/rhesus protein (AMT/MEP/Rh) family [49,50]. In *Arabidopsis thaliana*, four homologs from the AMT family (AMT1;1, AMT1;2, AMT1;3, and AMT1;5) and one homolog from the MEP subfamily (AMT2;1) are expressed in roots and AMT2;1 localizes at the plasma membrane [51,52]; these function mainly in the root-to-shoot translocation of ammonium [53]. More ammonium was accumulated in the shoots of the *vha-a2 vha-a3* double mutant than in those of the wild type; therefore, we measured the metabolism and long-distance transport of nitrogen between the shoots and roots. In roots, the relative expression of *NRT1.5* was downregulated and that of *NRT1.8* was upregulated (Figure 4a,b), which increased the transport of NO_3_^−^ from the shoots to the roots in the mutant (Figure 3a). Moreover, NR activity was notably induced (Figure 3b). However, there was a significant decrease in ammonium content in the roots of the *vha-a2 vha-a3* mutant (Figure 3c), and there was no significant difference in GS activity between the two lines (Figure 3d). The relative expression of *AMT2;1* in roots was upregulated two-fold, and xylem sap NH_4_^+^ content was 66% higher than that of Col-0 (Figure 4c,d), leading to more NH_4_^+^ to be transported from the roots to the shoots. Similarly, the root/shoot ratio in NH_4_^+^ decreased significantly in the *vha-a2 vha-a3* mutant compared to that of the wild type (Figure S2B), indicating a greater proportion of ammonium being allocated to the leaves in the double mutant than in the wild type. Both the reduced ammonium metabolism in the shoots and the higher transport of ammonium from roots to shoots affected the accumulation of ammonium in the shoots, thereby aggravating NH_4_^+^ poisoning in *vha-a2 vha-a3* double mutant plants.

### 2.5. Impaired Tonoplast V-ATPase Activity Influences Ion Homeostasis

**Figure 5:**
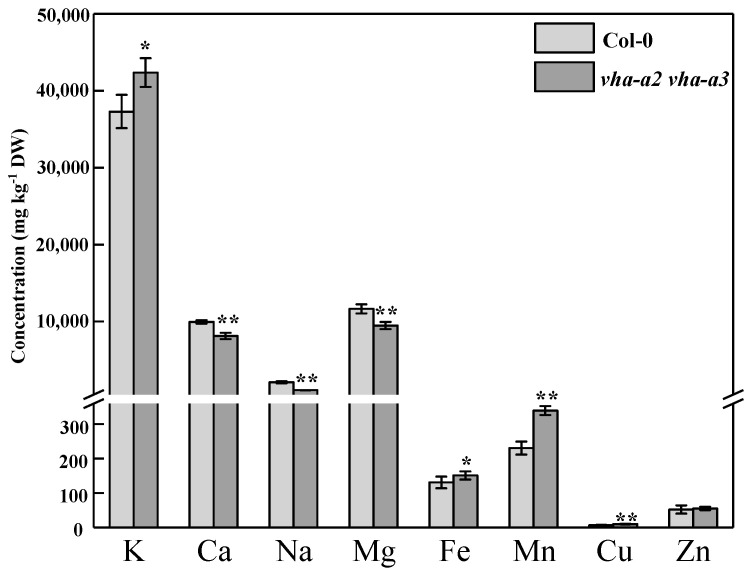
Impaired tonoplast V-ATPase activity prejudiced ion homeostasis. The concentration of K^+^, Ca^2+^, Na^+^, Mg^2+^, Fe^2+^, Mn^2+^, Cu^2+^, and Zn^2+^ in the shoots of wild type and *vha-a2 vha-a3* mutant. Asterisks (*) and (**) indicated significant differences at *p* < 0.05 and *p* < 0.01, respectively.

We assessed whether ion content was disrupted in plants in the absence of tonoplast V-ATPase activity. Cations in rosette leaves, namely potassium (K^+^), calcium (Ca^2+^), magnesium (Mg^2+^), sodium (Na^+^), iron (Fe^2+^), manganese (Mn^2+^), copper (Cu^2+^), and zinc (Zn^2+^), were extracted from four-week-old plants and analyzed by inductively coupled plasma-mass spectrometry (ICP-MS) (Figure 5). Compared to the wild type, the content of Ca^2+^, Mg^2+^, and Na^+^ decreased, and the content of Fe^2+^, Mn^2+^, and Cu^2+^ increased in the mutant, indicating that ion homeostasis was disturbed in plants in the absence of tonoplast proton pumps, which was correlated to decreased plant growth and premature senescence.

### 2.6. Growth Retardation is Alleviated and Senescence is Delayed by Supplementing K^+^ to the Mutant

Reduced leaf growth of the *vha-a2 vha-a3* double mutant under control conditions was accompanied by leaf etiolation; these symptoms were similar to those caused by calcium deficiency in the *cax1 cax3* double mutant that lacked two vacuolar Ca^2+^/H^+^ antiporters [54,55]. To address the lower calcium content in the double mutant, additional doses of Ca^2+^ (0.5 mM, 2.5 mM, 5 mM) were supplied to the *vha-a2 vha-a3* double mutant during the four-week hydroponic cultivation. The yellowing phenotype of the mutant was not restored after addition of any of the three concentrations of Ca^2+^. Furthermore, plants were markedly smaller than those of the wild type (Figure S3A).

The effect of K^+^ on plants was examined by supplying different doses of K^+^ (2 mM, 4 mM, 6 mM, 8 mM, 10 mM, 15 mM, and 20 mM) to the plants. The difference in plant size between the *vha-a2 vha-a3* double mutant and the wild type gradually diminished, and the symptoms of leaf chlorosis were alleviated with increasing external K^+^ concentrations from 2 mM to 10 mM (Figure S3B). However, these effects were reduced when the concentration of K^+^ in the culture solution was 15 mM and 20 mM. Compared with those of the 2 mM K^+^ supplementation, the changes in the wild type were not significant after the addition of different K^+^ doses. The content of NO_3_^−^, NH_4_^+^, and chlorophyll decreased within the potassium concentration range of 2 mM to 10 mM, and slightly increased at 15 mM and 20 mM (Figure S4A–C).

**Figure 6:**
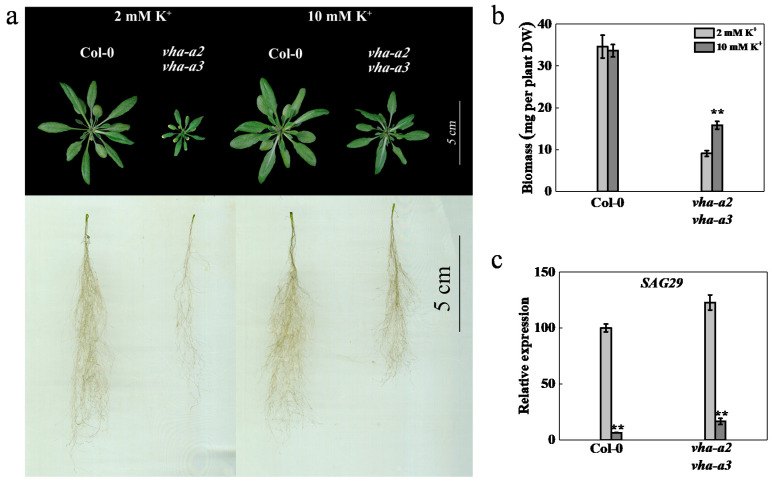
The growth performance of *vha-a2 vha-a3* mutant was improved with the additional K^+^ supplied. (**a**) the phenotype of wild type and *vha-a2 vha-a3* mutant with additional K^+^ supplied. Pictures show four-week-old plants with different K^+^ supply levels. (**b**) the biomass of Col-0 and *vha-a2 vha-a3* mutant was determined. (**c**) gene expression levels of *SAG29* in four-week-old leaves of wild type and *vha-a2 vha-a3* mutant with different levels of K^+^. Wild type with an additional supply of 2 mM K^+^ was set to 100% and error bars were defined as S.E. of *n* = 3 biological replicates. Pictures show four-week-old plants with different K^+^ supply levels. Error bars in (**b**) indicate S.D. of *n* = 3 technical replicates. Asterisks (**) indicate significant differences at *p* < 0.01.

The *vha-a2 vha-a3* double mutant demonstrated increased shoot and root biomass after supplying 10 mM external K^+^ when compared to that in the treatment with 2 mM external K^+^ (Figure 6a). The biomass of mutant plants was increased by 74% and relative expression levels of *SAG29* were decreased by 87% at potassium concentrations from 2 mM to 10 mM (Figure 6b,c). Concomitantly, the leaf area, total root length, root surface area, root volume, and number of root tips and forks were enhanced in wild type and mutant plants when grown in 10 mM K^+^ (Figure S5); a much greater growth difference was observed in the *vha-a2 vha-a3* double mutant, indicating that exogenous potassium could alleviate the growth inhibition and delay senescence to some extent in double mutant plants.

### 2.7. Nitrogen Absorption is Promoted and Ammonium Accumulation is Reduced with Additional K^+^ Provision to the Mutant

**Figure 7:**
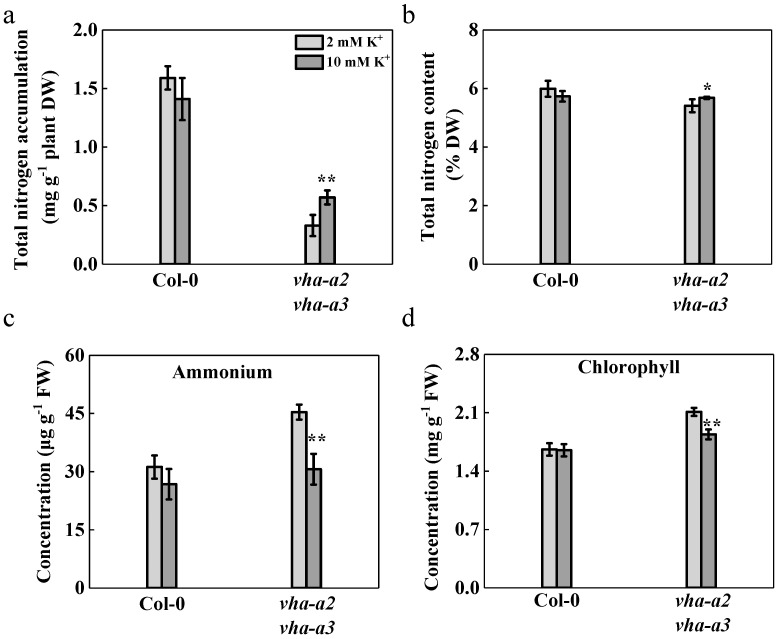
Promotion of nitrogen absorption and reduction of ammonium accumulation with an additional K^+^ supply in mutant. The total nitrogen accumulation (**a**), total nitrogen content (**b**), ammonium concentration (**c**), and chlorophyll concentration (**d**) were measured with different K^+^ doses for wild type and *vha-a2 vha-a3* mutant grown in hydroponics for four-week-old plants. Error bars indicated S.D. of *n* = 3 technical replicates. Asterisks (*) and (**) indicate significant differences at *p* < 0.05 and *p* < 0.01, respectively.

It has been long noticed that K^+^/NO_3_^−^ absorption and transport in vascular plants are somehow coordinated, playing a critical role in promoting NO_3_^−^ absorption [31,32,33,34]. Total nitrogen accumulation and total nitrogen content of the double mutant increased by 73% and 5%, respectively, in 10 mM K^+^ treatment compared with those in 2 mM K^+^ treatments; this, in turn, improved the growth of mutant plants (Figure 7a,b). In contrast, the biomass and nitrogen absorption of the wild type showed no significant difference between treatments with 2 mM and 10 mM K^+^ (Figure 6b and Figure 7a,b).

Surprisingly, ammonium and chlorophyll content in the shoots of mutant plants was reduced by 32% and 13%, respectively, after adding 10 mM potassium in the nutrient solution (Figure 7c,d and Figure S4B,C). Increased nitrogen accumulation and decreased ammonium content in shoots resulting from additional K^+^ supplementation helped to restore the growth performance and delayed the senescence, but the specific mechanisms involved in these processes remain uncertain and warrant further studies.

## 3. Discussion

The two proton pumps in plant tonoplast, vacuolar H^+^-ATPase and vacuolar H^+^-PPase, are highly abundant tonoplast proteins that play important roles in plant growth and development [1,5]. Both proton pumps energize the massive fluxes of ions and metabolites that are required for vacuolar function throughout the plant life cycle [4]. To elucidate the role of V-ATPase in the growth of plants, we selected an *Arabidopsis thaliana* line with loss of V-ATPase in the tonoplast, *vha-a2 vha-a3*, which lacks the two tonoplast-localized isoforms of the membrane-integral V-ATPase subunit VHA-a.

The mutant plants are viable but present severe growth inhibition and premature aging, including reduced biomass, leaf area, total root length, root surface area, root volume, and the number of root tips and forks. Nitrate, as the main source of nitrogen, is partitioned between the cytoplasm and the vacuole after its uptake by the root [21]. Vacuolar NO_3_^−^ storage and its subsequent remobilization are a central feature of the plant nitrogen economy [4]. The reduced vacuolar uptake capacity can lead to increased cytosolic NO_3_^−^ levels, which in turn stimulate nitrate assimilation, a process that requires a substantial electron flow via either ferredoxin or NAD(P)H [56].

Compared to that of the wild type, the content of ammonium was increased by 31% and the chlorophyll content was higher in mutant line, a condition which is potentially toxic to plants. Chlorophyll content is an indicator of ammonium stress and its content increases under mild ammonium stress in *Arabidopsis thaliana* [41]. Our data revealed that the NO_3_^−^ metabolism was blocked when V-ATPase activity was absent, likely due to increased vacuolar pH. Consequently, the vacuoles cannot retain optimal acidity and intracellular environment conducive to normal metabolism of nitrate in cells.

Interestingly, in *Arabidopsis thaliana*, the lack of tonoplast V-ATPase activity could affect the long-distance transport of nitrogen between shoots and roots. In roots, the amount of nutrients destined for long-distance transportation is determined by the activity of transporters that load the xylem vessels, while in shoots, transporters unloading into the xylem can increase the overall root-to-shoot translocation [44,45,46,57,58]. The relative expression of *NRT1.5* was downregulated and that of *NRT1.8* was upregulated in mutant plants, which caused more NO_3_^−^ to accumulate and induced a notable increase in NR activity in the roots. However, surprisingly, a notable decrease in ammonium content was detected in the roots of mutant plants, but there was no difference in GS activity between the two lines. A previous study has revealed that *AMT2;1*, which encodes a high-affinity ammonium transporter, functions mainly in the translocation of ammonium from roots to shoots [53]. The relative expression of *AMT2;1* was upregulated in the mutant, and xylem sap NH_4_^+^ content was about twice as high as that in the wild type. As a result, more ammonium was transported from roots into the shoots, which in turn caused the accumulation of ammonium, aggravating the ammonium toxicity in the shoots of *vha-a2 vha-a3* double mutant plants.

The proton pumps in the tonoplast affect the vacuolar transport capacities and are considered crucial for translocation of harmful ions from the cytoplasm into the vacuoles, reduction of excessive ion toxicity to cells, and stabilization of ion homeostasis in the cell [59], as well as for the regulation of metabolite homeostasis, plant growth, and plant adaptation to abiotic stress conditions [7,12,17,18,19,20]. In the mutant, the content of Ca^2+^, Mg^2+^, and Na^+^ was decreased, and that of Fe^2+^, Mn^2+^, and Cu^2+^ was increased. The disturbance of ions in cells may be caused by a decrease in the electrochemical potential gradient between the tonoplast and cytoplasm, which leads to the alkalization of vacuoles, thereby affecting the activity of various ion transporters and channel proteins in the tonoplast and plant growth.

The impaired growth of the *vha-a2 vha-a3* double mutant under normal growth conditions was accompanied by smaller leaf area and etiolated leaves. These are similar to the symptoms of calcium deficiency also found in the *cax1 cax3* double mutant that lacks two vacuolar Ca^2+^/H^+^ antiporters [54,55]. Our results showed a reduced content of Ca^2+^ in the mutant. We aimed to investigate whether the growth inhibition of mutant plants was caused by Ca^2+^ deficiency. To explore this aspect, we supplemented the hydroponic nutrient solution with different concentrations of Ca^2+^; the growth limitation of mutants did not abate, indicating that Ca^2+^ deficiency was not the major factor inhibiting plant growth. Considering the fact that NO_3_^−^ is abundant in plants and its absorption and transport are believed to be accompanied by K^+^ [33,34], we supplied different K^+^ doses to the double mutant. The growth retardation of mutants was alleviated to some extent by 2 mM to 10 mM potassium supplementation—biomass and leaf area increased, and the total root length, root surface area, root volume, and the number of root tips and forks was enhanced. However, the double mutants remained smaller than the wild type. The activity of V-PPase, another main proton pump in the tonoplast, was increased six-fold by potassium supplementation [38], which can explain the partial rescue of the plant phenotype at high potassium concentrations; however, the growth phenotype indicated that V-PPase cannot completely replace and compensate for the functions of V-ATPase. The positive effect of potassium supplementation was reduced when the concentration of K^+^ in the culture solution was 15 mM and 20 mM, which exposed the negative effect of high potassium concentration on plant growth. The increase in external K^+^ to 10 or 20 mM KCl significantly affected plant growth, leading to a severe growth reduction [60]. Additionally, the acquisition rates of K^+^ and NO_3_^−^ are often positively correlated and enhance each other [33,34]; potassium supplementation increased total nitrogen accumulation and reduced the content of ammonium, delaying senescence and death of rosette leaves. Compared with those of the 2 mM K^+^ treatment, the changes in the wild type were not significant after potassium addition likely due to homeostasis that was established in its cells, and the addition of potassium had no further effect on plant growth. Therefore, potassium supplementation alleviates the growth inhibition. One possible explanation for this is that the interactions between NO_3_^−^ and K^+^ may aid in the absorption of nitrogen by plants, leading to increased biomass of mutants [61]. Moreover, potassium may alleviate ammonium toxicity in mutants; this notion is supported by a previous work showing that an increased K^+^ supply or a restoration of K^+^ transport can rapidly alleviate NH_4_^+^ toxicity [39,40].

In conclusion, we revealed the important role of V-ATPase activity in growth of *Arabidopsis thaliana* plants. An impaired growth and excessive ammonium accumulation were observed in the *vha-a2 vha-a3* double mutant. Interestingly, the limited growth can be alleviated by adding additional potassium. This study provided a new insight into the relationship between vacuolar H^+^-ATPase, growth, and ammonium accumulation. Concomitantly, we revealed the role of potassium in mitigating ammonium stress and, via adjustment of the activity of tonoplast V-ATPase and the content of potassium, for regulation of ammonium metabolism and growth.

## 4. Materials and Methods

### 4.1. Plant Materials and Growth Conditions

The wild type *Arabidopsis thaliana* line Columbia-0 (Col-0) was used as a control for the tonoplast V-ATPase double mutant (*vha-a2 vha-a3*). The *vha-a2 vha-a3* double mutant was kindly provided by Dr. M. Krebs (Heidelberg Institute for Plant Science, University of Heidelberg, Germany). The mutant was identified in the F_2_ generation derived from a cross between homozygous *vha-a2* (SALK_142642) and *vha-a3* (SALK_29786) individuals [4]. The experiments were carried out in a phytotron (MGC-800HP-2; Bluepard Test Equipment Company, Shanghai, China) at the Hunan Agricultural University, Changsha, China. The phytotron was set at a 16-h light/8-h dark photoperiod, 70% relative humidity, and constant temperature of 22 °C. Seeds were sown in a matrix consisting of vermiculite and perlite at a ratio of 3:2, and allowed to germinate and grow for 10 days [62,63]. Subsequently, the seedlings at the two-leaf stage were transferred and grown hydroponically in 600 mL plastic pots covered with a black film on the outside, as per methods described by Gong et al. [64]. Nine plants were grown in each plastic pot. To eliminate edge effect, the positions of the pots were interchanged when refreshing the media. The nutrient solution (pH = 5.8) provided to plants was replaced every five days, as described in Han et al. [65]. The nutrient solution used for both lines consisted of 1.25 mM KNO_3_, 0.625 mM KH_2_PO_4_, 0.5 mM Ca(NO_3_)_2_·4H_2_O, 0.5 mM MgSO_4_, 0.025 mM Fe-EDTA, and 0.25 mM micronutrients (stock solution concentrations: 70 mM H_3_BO_3_, 14 mM MnCl_2_, 1 mM ZnSO_4_, 0.5 mM CuSO_4_, and 0.2 mM Na_2_MoO_4_).

When treated with potassium, the seedlings were transplanted and grown in the nutrient solution mentioned above supplemented with different concentrations of potassium (2 mM, 4 mM, 6 mM, 8 mM, 10 mM, 15 mM, and 20 mM) for 30 days under constant growth conditions. To examine the effect of Ca^2+^, the seedlings were transplanted and grown in the nutrient solution mentioned above supplemented with Ca^2+^ at concentrations of 2.5 mM and 5 mM for 30 days under constant growth conditions.

### 4.2. Measurement of Leaf Rosette Areas

Whole rosette leaves were collected from individual *Arabidopsis thaliana* seedlings grown for four weeks. Leaf area was measured using a leaf area meter (CI-202 Laser Area Meter; CID Bio-Science, Camas, WA, USA).

### 4.3. Measurement of Root Configuration

The roots were sampled from individual *Arabidopsis thaliana* seedlings grown for four weeks. They were spread completely in 1000 cm^3^ (25 cm by 20 cm by 2 cm) transparent plastic containers filled with distilled water. Root surface was washed to remove impurities, and the total root length, root surface area, root volume, and the number of root tips and forks were determined using WinRHIZO (EPSON Expression 11000XL, Japan).

### 4.4. Determination of Nitrogen Content

Whole seedlings of *Arabidopsis thaliana* grown hydroponically for four weeks were sampled and oven-dried at 105 °C for 30 min, followed by drying at 65 °C to a constant weight. The samples were digested with H_2_SO_4_-H_2_O_2_ (5 mL H_2_SO_4_) at 350 °C and analyzed using an AA3 continuous-flow auto-analyzer [65]. Nitrogen concentration was calculated using the following formula: Total N = [N] × biomass.

### 4.5. Assays for NO_3_^−^ Content Estimation

Fresh plants were divided into shoots and roots as per methods described by Han et al. [65], and nitrate was extracted from tissue samples (shoot: 1 g; root: 0.5 g). The samples were soaked in deionized water and heated in a boiling water bath for 30 min. Then, 0.1 mL of the sample solution was mixed well with 0.4 mL of 5% salicylic acid-H_2_SO_4_ solution (5 g salicylic acid dissolved in 100 mL H_2_SO_4_) and allowed to react at room temperature for 20 min. Subsequently, 9.5 mL of 8% sodium hydroxide solution was added to the mixture and left to cool to room temperature. The content of NO_3_^−^ was measured spectrophotometrically at 410 nm following the method reported by Cataldo et al. [66].

### 4.6. Determination of NH_4_^+^ Content

To determine the content of NH_4_^+^, shoots and roots were sampled as described above and extracted with deionized water for 30 min. NH_4_^+^ content was measured using indophenol blue colorimetry at an absorbance wavelength of 630 nm [67].

### 4.7. Determination of the Activities of Nitrate Reductase (NR) and Glutamine Synthetase (GS)

The method for determining NR activity was as previously described [68,69], with slight modification. Fresh plants were separated into shoot (0.5 g) and root (0.5 g) samples. The samples were washed with deionized water and wiped dry, snap-frozen in liquid nitrogen at −196 °C for 30 min, and stored at −80 °C for determination of enzymatic activity. A small amount of quartz sand and 5 mL phosphate buffer (0.1 M, pH = 7.5) were added to a pre-frozen mortar, and the frozen samples were ground to homogeneity using a chilled pestle. The homogenates were centrifuged at 2000× *g* for 15 min at 4 °C. The supernatant fluid was used to determine the activity of NADH-dependent NR. The reaction mixture consisted of 0.4 mL supernatant, 0.1 M KNO_3_, and 3 mM NADH. The reaction was terminated after 30 min at 25 °C by adding 1% sulfanilamide and α-naphthylamine. The amount of reaction product was measured at 540 nm using a UV-2600 spectrophotometer (Shimadzu Corp., Kyoto, Japan).

To measure GS activity, frozen plant samples (shoot: 0.5 g; root: 0.5 g) were ground to obtain homogeneous samples as presented above with 3 mL 50 mM Tris-HCl buffer (pH = 8.0), containing 2 mM dithiothreitol, 2 mM Mg^2+^, and 0.4 M sucrose. The homogenate was centrifuged at 10,000× *g* for 10 min at 4 °C. The supernatant was examined for GS activity according to the method described by Zhang et al. [70].

### 4.8. Chlorophyll Content Assays

Fresh rosette leaves (0.2 g) were sampled and extracted in 10 mL absolute ethanol: Acetone (1:1) for 48 h in the dark at 4 °C [71]. The absorbance of the extract was measured at 663 and 645 nm using a UV-VIS spectrophotometer (UV-2600, Shimadzu, Kyoto, Japan) to estimate total chlorophyll content.

### 4.9. RNA Extraction and qRT-PCR Assay

The seedlings were grown under normal conditions for 30 days after which shoots (0.1 g) and roots (0.1 g) were harvested for RNA analysis. Total RNA was extracted with 1 mL TRIzol reagent (Invitrogen, Carlsbad, CA, USA), precipitated with an equal volume of isopropanol, washed with 75% ethanol, and dissolved in RNAase-free water. The first-strand cDNA was synthesized from total RNA using the PrimeScript reverse transcription (RT) reagent kit (TaKaRa, Shiga, Japan) in accordance with the manufacturer’s instructions. The relative gene expression was determined using the SYBR Green RT-PCR kit (TaKaRa) with a pair of gene-specific primers. The RT-PCR analysis was performed using the StepOnePlus™ Real-Time PCR Instrument (StepOnePlus™, Life Technologies Holdings Pte Ltd., Singapore) following the manufacturer’s instructions. The thermal cycles were as follows: 95 °C for 3 min, followed by 40 cycles of 95 °C for 10 s and 60 °C for 30 s. A melt curve analysis was performed to ensure the primer gene specificity as follows: 95 °C for 15 s, 60 °C for 1 min, 60–95 °C for 15 s. The primers used in this analysis are presented in Supplementary Table S1, and the expression data were normalized to those of Actin2 [72,73].

### 4.10. Determination of Ammonium in Xylem Sap

Seedlings were gathered and stripped of leaves, and the stems were cut about 1 cm from the root and shielded from light. The xylem sap was collected with capillaries within 2 h of cutting. Each plant was covered with one capillary for xylem sap collection. Three biological replicates were prepared for the double mutant and wild type. The xylem sap was collected from 20 plants; the total collected volume was approximately 50 μL per replicate [74]. Ammonium content was determined as described by Santoni et al. [67].

### 4.11. Determination of Cation Content

The method for determining cation content was a slightly modified protocol used in a previous study [75]. Shoots of the seedlings that were hydroponically grown for four weeks were collected and oven-dried at 105 °C for 30 min and then at 65 °C to constant weight. Dried samples were ground into a fine powder and about 100 mg of sample was mixed with 5 mL HNO_3_ and heated in boiling water for 2 h. The volume of the extracting solution was adjusted to 50 mL with deionized water after cooling. The cation content was measured by ICP-MS (ELAN DRC-e, Perkin Elmer, Shelton, CT, USA).

### 4.12. Statistical Analyses

SPSS 17.0 (Ibm Corp., Chicago, IL, USA) was used for one-way analysis of variance (ANOVA) and Tukey’s honestly significant difference (HSD) multiple comparison tests. All experiments were conducted using a completely randomized design. Three samples were prepared per treatment, with two technical replicates per sample. Differences between the wild type and *vha-a2 vha-a3* double mutant and/or between different treatments were considered statistically significant for *p* < 0.05.

### 4.13. Accession Numbers

Sequence data for this article were obtained from the GenBank/EMBL data libraries under the GenBank accession numbers NM 112,764 (*Actin2*), NM 129,385 (*AMT2;1*), NM 102,980 (*NRT1.5*), NM 118,288 (*NRT1.8*), and NM 121,320 (*SAG29*). The GenBank accession numbers for each of the gene mentioned and sequences were obtained from the TAIR (https://www.arabidopsis.org/).

## Supplementary Materials

The following are available online at https://www.mdpi.com/1422-0067/22/1/2/s1, Figure S1: The leaf area and root configuration of wild type and the *vha-a2 vha-a3* double mutant, Figure S2: Chlorophyll concentration and NH4+ root/shoot ratio (R/S) of wild type and *vha-a2 vha-a3* mutant, Figure S3: The phenotype of wild type and *vha-a2 vha-a3* mutant with different Ca^2+^ and K^+^ supply levels; Figure S4: Nitrogen metabolites in wild type and *vha-a2 vha-a3* mutant with different potassium concentrations applied, Figure S5: The leaf area and root configuration of wild type and the *vha-a2 vha-a3* mutant with different K^+^ doses applied; Table S1: Primers of the genes targeted in the qRT-PCR assays.
