# Supplementary material for: Ammonium Accumulation Caused by Reduced Tonoplast V-ATPase Activity in Arabidopsis thaliana
Source: Int J Mol Sci. 2020 Dec 22;22(1):2. doi: 10.3390/ijms22010002 (PMC7792577; doi:10.3390/ijms22010002)
Supplement: Supplementary file 1 [file ijms-22-00002-s001.pdf]

International Journal of *Molecular Sciences*

MDPI

Ammonium Accumulation Caused by Reduced Tonoplast V-ATPase Activity in *Arabidopsis thaliana*

Guihong Liang[1], Haixing Song[1], Yan Xiao[1], and Zhenhua Zhang[1*]

[1]Southern Regional Collaborative Innovation Center for Grain and Oil Crops in China, College of Resources and Environmental Sciences, Hunan Agricultural University, Changsha, 410128, China

ghliang1119@163.com (G.L.); 18229474886@163.com (H.S.); xiaoxiaoy8432@163.com (Y.X.)

*Correspondence: zhzh1468@163.com (Z.Z.)

Table S1 Primers of the genes targeted in the qRT-PCR assays.

Gene ID	GenBank accession	Primer	Sequence(5'-3')	Method
AT2G38290	NM129385	AMT2;1-F	CGGGAAAGATAGAATAACAAAATGG	Real-time qPCR
		AMT2;1-R	ATTGCTCCGATGACAGAAGG	
AT1G32450	NM102980	NRT1.5-F	TGTCATTGGACTTTCATCGC	
		NRT1.5-R	CCCACAACCTCTTGGTCTAATC	
AT4G21680	NM118288	NRT1.8-F	GGCTTCAGATTCTTGGATAG	
		NRT1.8-R	AACCACAGAGTAGAGGATGG	
AT5G13170	NM121320	SAG29-F	GCCACCAGGGAGAAAAGG	
		SAG29-R	CCACGAAATGTGTTACCATTAGAA	
AT3G18780	NM112764	Actin2-F	CCACGAAATGTGTTACCATTAGAA	
		Actin2-R	TTTCCCGCTCTGCTGTTGT	

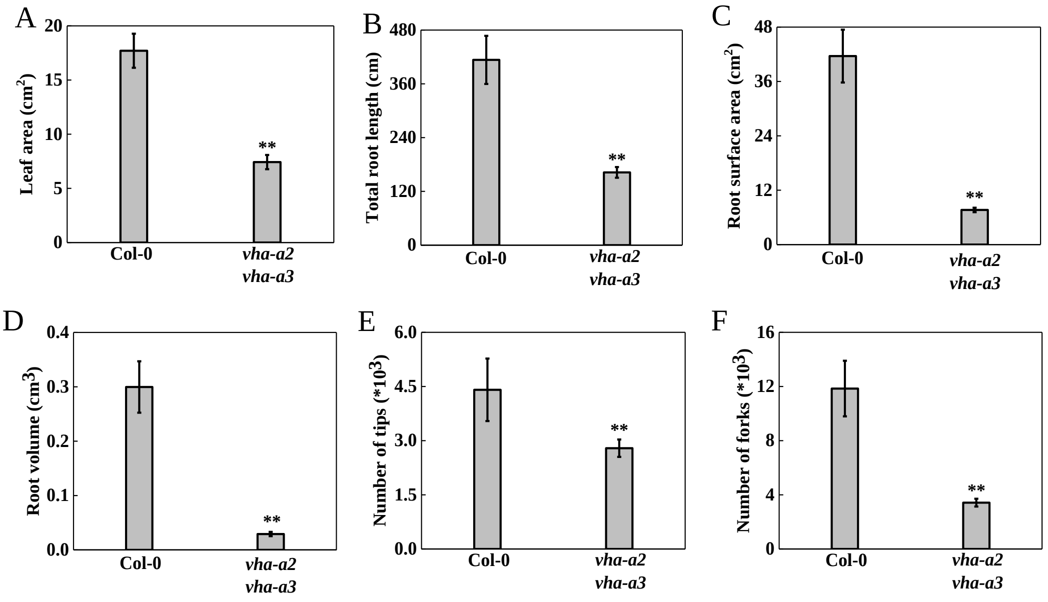

Figure S1 The leaf area and root configuration of wild type and the *vha-a2 vha-a3* double mutant. The rosette area (A), total root length (B), root surface area (C), root volume (D), the number of tips (E) and forks (F) were determined for Col-0 and *vha-a2 vha-a3*. Error bars represent S.D. of n = 3 biological replicates. Asterisks (**) indicate significant differences at $p < 0.01$.

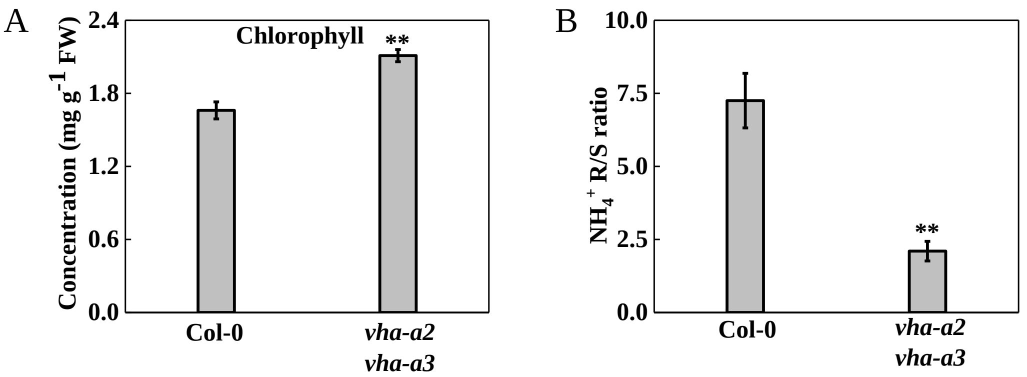

Figure S2 Chlorophyll concentration and NH_4^+ root/shoot ratio (R/S) of wild type and *vha-a2 vha-a3* mutant. The seedlings were grown for four-week-old under normal condition. Error bars were defined as S.D. of n = 3 biological replicates. Asterisks (**) indicate significant differences at $p < 0.01$. FW, Fresh weight.

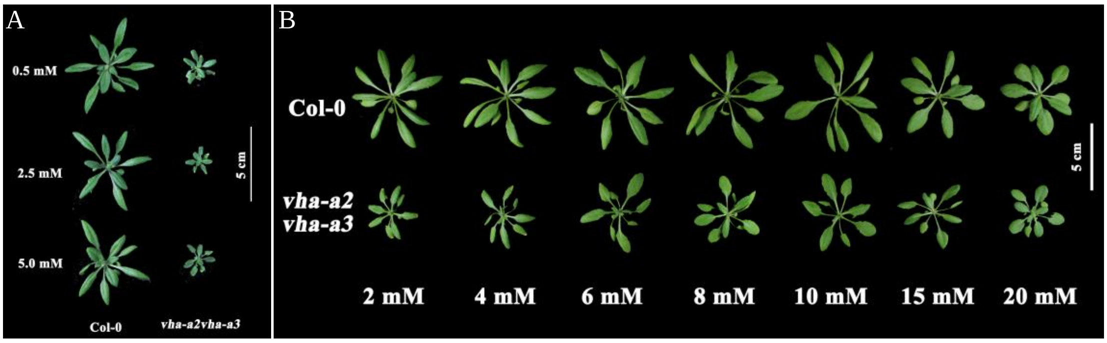

Figure S3 The phenotype of wild type and *vha-a2 vha-a3* mutant with different Ca^{2+} and K^{+} supply levels. (A) The phenotype of wild type and *vha-a2 vha-a3* mutant with additional Ca^{2+} supplied. (B) The phenotype of wild type and *vha-a2 vha-a3* mutant with additional K^{+} supplied. Pictures show four-week-old plants with different Ca^{2+} and K^{+} supply levels.

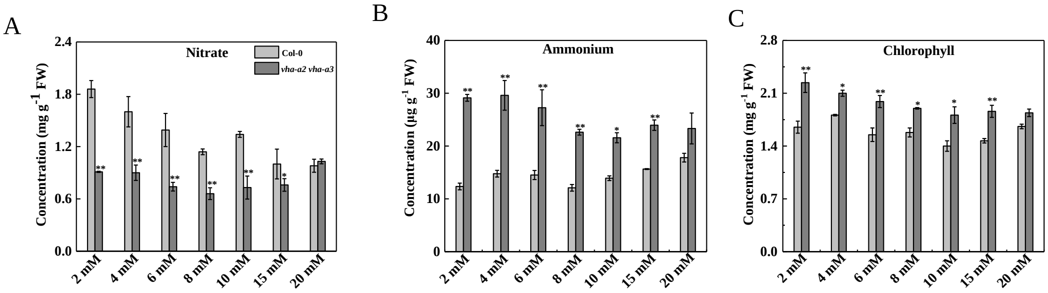

Figure S4 Nitrogen metabolites in wild type and *vha-a2 vha-a3* mutant with different potassium concentrations applied. The concentration of nitrate (A), ammonium (B), and chlorophyll (C) were determined with different K^{+} supply levels from shoots. Error bars were defined as S.D. of n = 3 technical replicates. Asterisks (*) and (**) indicate significant differences at $p < 0.05$ and $p < 0.01$, respectively.

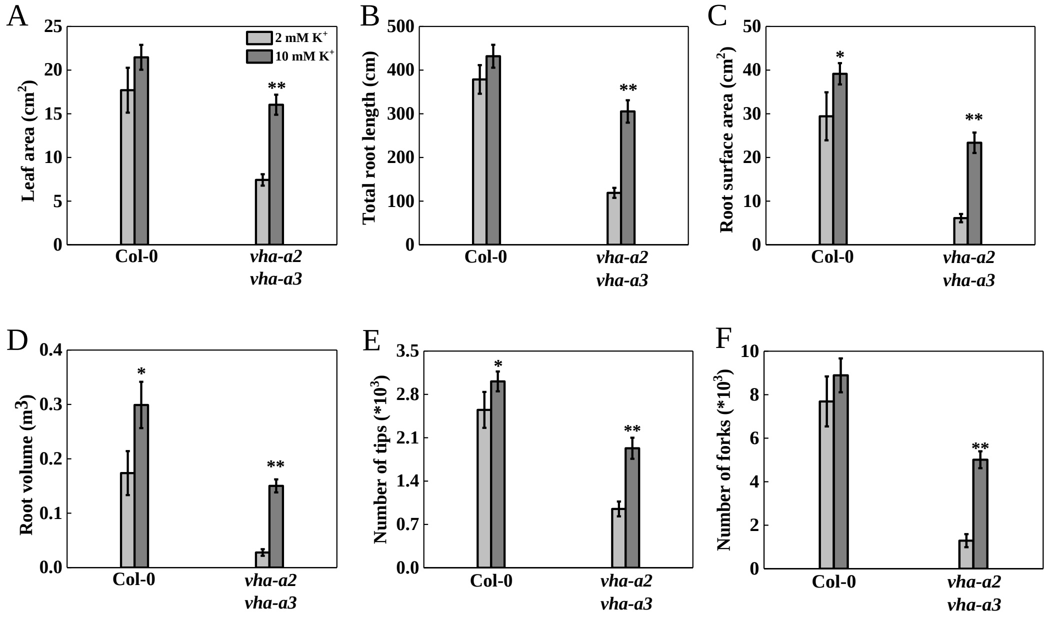

Figure S5 The leaf area and root configuration of wild type and the *vha-a2 vha-a3* mutant with different K^+ doses applied. The rosette area (A), total root length (B), root surface area (C), root volume (D), the number of tips (E) and forks (F) were determined for Col-0 and *vha-a2 vha-a3* mutant with different K^+ doses. Error bars represent S.D. of n = 3 biological replicates. Asterisks (*) and (**) indicate significant differences at $p < 0.05$ and $p < 0.01$, respectively.
